# IMMERSIVE VIRTUAL REALITY TO ASSESS UNILATERAL SPATIAL NEGLECT IN STROKE PATIENTS: A PRELIMINARY STUDY

**DOI:** 10.2340/jrm.v57.41195

**Published:** 2025-01-03

**Authors:** Chloé SAUVAGE, Pierre CHAULET, Luana Rivas LOPEZ, Johanne GARBUSINSKI, Pierre CABARAUX, Zachary DUVIGNEAUD, Stéphane BAUDRY

**Affiliations:** 1 Laboratory of Applied Biology and Research Unit in Applied Neurophysiology (LABNeuro), Faculty of Human Movement Sciences, Université Libre de Bruxelles, Brussels; 2 Department of Neurorehabilitation, Hôpital Erasme-HUB, Brussels; 3 Department of Physiotherapy, Hôpital Erasme-HUB, Brussels, Belgium

**Keywords:** neglect, stroke, cancellation test, virtual reality

## Abstract

**Objectives:**

The conventional test to detect unilateral spatial neglect (USN) is the Bells Test performed in a paper-and-pencil format. While several studies showed immersive virtual reality (VR) tests may provide greater sensitivity in revealing the presence of USN using visual scanning tasks, none has investigated the Bells Test in VR. This study compares the Bells Test performed in paper-and-pencil format (PP) and in VR in conventional (CVR) and ecological (EVR) format, which differ by the size of the display, in stroke patients.

**Design:**

Cross-sectional study.

**Setting:**

Stroke patients.

**Participants:**

A convenience sample of 32 stroke patients.

**Interventions:**

VR assessments were performed using an immersive system with a head-mounted display. In CVR, the Bells Test is reproduced in the same format as PP (A4 sheet), while in EVR, the targets are displayed in a wider space corresponding to a hemisphere of 1-m radius.

**Results:**

The number of cancelled targets out of 35 was 32.5 (3.5) for PP, 33 (4) for CVR, and 34 (2) for EVR (mean [SD]), with a significant difference between PP and EVR (*p* < 0.05). The time to complete the Bells Test was 186 (69) s for PP, 184 (65) s for CVR, and 170 (58) s for EVR, without differences between modalities (*p* > 0.05). Bells Tests in the 3 modalities revealed the presence of USN, except for 1 patient in EVR.

**Conclusion:**

VR assessment of USN could be used in the same way as conventional cancellations tests. Moreover, VR could provide additional information on the type of USN through the different testing modalities available.

## LAY ABSTRACT

This study aimed to compare the Bells Test performed in paper-and-pencil format and in virtual reality format (2 different formats, conventional and ecological, which differ by the size of the display) in stroke patients. A convenience sample of 32 stroke patients, divided into 2 groups, with and without unilateral spatial neglect, participated in this study. Results showed that the time required to complete the Bells Test did not differ between the 3 modalities in each subgroup. The reliability between the 3 modalities was high for global and lateralized task performance. No difference between modalities for global and lateralized task performance was observed except between the paper-and-pencil format and the ecological format in no-neglect patients. These findings show that the virtual reality Bells Test is easy to use, very well accepted by patients, does not induce any relevant side effects in stroke patients and assesses both near and far extrapersonal space.

Unilateral spatial neglect (USN) is a disabling condition frequently occurring after stroke. USN is characterized by the inability to orientate or respond to or report a stimulus appearing on the contralesional side, even if the patient can move their head and eyes (1). USN has a well-established negative impact on functional recovery, community reintegration, and quality of life (3). Patients with USN present a wide range of functional spatial deficits, such as bumping into objects when walking, reading only 1 side of a page, sentence, or word, shaving only 1 side of their face, and eating food from only 1 side of the plate (4). USN is characterized by different anatomo-clinical subtypes (visual, auditory, somatosensory, motor, allocentric, egocentric, and representational neglect) that may be associated or dissociated (5).

Clinically, severe USN is easily observable, whereas mild or moderate USN often goes undetected. Considering the negative impact of USN on functional recovery (6), the use of sensitive USN detection is essential for those patients. As a first assessment, USN is typically evaluated using a paper-pencil (PP) test (7), with cancellation tests being the most frequently used. In these tests, patients are required to cross out target objects sometimes embedded in distractors (8). The other most frequently used PP tests are copy tests such as the line bisection test and the drawing object. In the line bisection test, patients are asked to indicate the middle of horizontal lines, while in object drawing patients are required to copy a picture. Among PP tests, copy tests are less sensitive than cancellation tests in identifying USN (9, 10). There are various cancellation tests, such as the Albert test (11) and the letter and star cancellation test (12). Yet, the most widely used is the Bells Test (13). This test includes numerous distractors with a random distribution of numerous targets (i.e., 35 bells and 280 distractors), making it more sensitive to detect mild to moderate USN (14). Conventional PP tests are easy to use and score, do not require much time and effort for the patients and are overall well supported by normative data. These particularities make them commonly used in clinical practice (16). However, the USN can affect the near-extrapersonal space (i.e., within arm’s reach) and/or the far-extrapersonal space (i.e., beyond the arm’s reach) (15), while the PP test assesses only USN of near-extrapersonal space and cannot fully evaluate the patient’s abilities in activities of daily living (17).

With the rapidly growing field of virtual reality (VR), USN diagnostic techniques could be enhanced beyond conventional methods. VR is a technology that simulates an artificially generated environment using software to reproduce a sensory, visual, sound, or haptic experience (18). VR can be immersive, providing a first-person view, through the use of a head-mounted display (HMD). Immersive VR induces a higher subjective sense of presence, which corresponds to the feeling that the VR environment represents a real situation rather than just a viewed video experience (19). This could be based on the fact that immersive VR requires the allocation of more brain and sensory resources for cognitive and motor control during a task, compared with a non-immersive VR system (20). Several studies have demonstrated the safe use of an immersive VR system in stroke patients, with no adverse effects and good patient acceptance of this type of system (4). Compared with PP tests, Tsirlin *et al*. (4) reported in a literature review that VR tests have greater sensitivity in revealing the presence of USN. Notably, all reported studies used visual scanning tasks to assess USN. Some studies assessed USN in both near-extrapersonal space and far-extrapersonal space (21–23). However, none of them used a virtual task based on the Bells Test.

The aim of the present study is to investigate the possibility to use immersive VR Bells Test to identify USN in stroke patients. Our main hypothesis is that VR bells is equivalent to the PP Bells Test. Our second objective is to investigate the possibility of assessing the near and far extrapersonal space by comparing 2 versions of the VR Bells Test. Finally, our third objective is to assess the degree of ease of use and patient satisfaction with the VR Bells Test, ensuring it does not cause cybersickness-type side effects.

## METHODS

### Participants

**Fig. 1:**
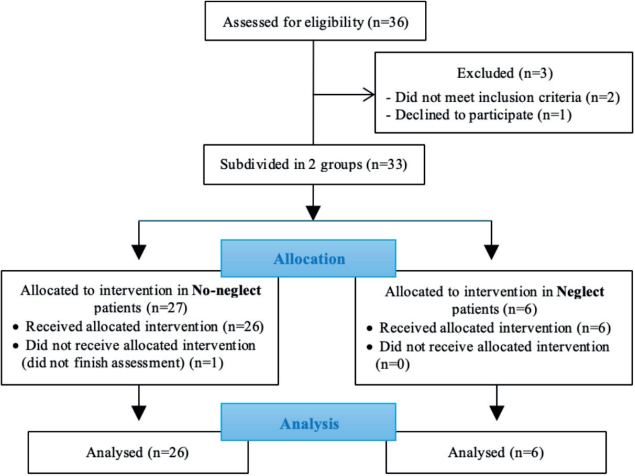
Patient flowchart from enrolment to analysis.

A sample of 32 stroke patients (mean age: 58.6 years, range 18–85; 23 men and 9 women) were recruited from the neurorehabilitation units of Erasme Hospital in Brussels in Belgium (Fig. 1). Inclusion criteria were the following: (*i*) haemorrhagic or ischaemic stroke within the past 2 years, (*ii*) no other neurological or psychiatric disease, (*iii*) aged between 18 and 85 years, (*iv*) no severe cognitive impairment (MMSE > 24) (24), (*v*) no difficulties in language comprehension, (*vi*) no visual and/or motor deficits hindering assessment.

Patients were divided into 2 groups: those without USN (*n* = 26) and those with USN (*n* = 6). This distinction was made based on clinical observations using the 10 items from the Catherine Bergego Scale (25) and/or scores from the neuropsychological evaluation, which included various items from the Test of Attentional Performance (26). Descriptive data on the sample are reported in Table I. Before participating in the study, each patient was given information on the study and was asked to give written consent to be included. The study was approved by the Ethics Committee of the Erasme Hospital (Belgium) and was conducted in accordance with good clinical practice guidelines and the latest version of the Declaration of Helsinki.

**Table I:** Demographic and clinical characteristics of patients

Group size (*n*)	32
Unilateral spatial neglect (left/right)	6 (5/1)
Age (mean ± SD)	58.6±14.0
Gender (males/females)	23/9
Stroke type (ischaemic/haemorrhagic)	27/5
Stroke side (left/right)	17/15
Weeks since stroke (mean±SD)	17.1±16.9

### Instrument

*Paper-and-pencil cancellation test.* The PP Bells Test was performed on an A4 sheet on which 315 stimuli are distributed. These include 35 bells and 280 distractors (birds, houses, clouds, etc.). The stimuli were arranged pseudo-randomly in 7 columns, 3 on the left, 1 in the middle and 3 on the right side of the sheet. Each column contained 5 bells. The sheet was centred exactly in front of the patient, and the patient had a maximum of 5 min to complete the task. The instructions were to cross out all the bells. According to the instructions for the Bells Test (13), the task ended when the patient told the experimenter that all the target objects stimuli were found, or when 5 min had elapsed. When the 35 bells were not found and the patient indicated that he/she had finished the test, the experimenter asked once: “Are you sure you have crossed out all the bells?” No further instructions were given during the task.

*Virtual reality cancellation test.* The VR system consists of a fixed computer running Virtualis software (Virtualis, Montpellier, France), a portable controller (Vive Controller, HTC, Taiwan) and an HMD (HTC Vive, HTC, New Taipei City, Taiwan) with a resolution of 2,160 × 1,200 pixels (full HD), a horizontal field of view of 110° and a frame rate of 90 Hz. The controller is perceived through the HMD as a laser. The patient can point to the various stimuli by orienting it towards them, and simply clicks a button when he/she wants to cross them out. Once the stimulus has been crossed out, it turns green. Reversal was possible by clicking a second time on the stimulus, which then returned to its original black colour.

The virtual cancellation task consisted of a duplicate of the PP in VR (35 bells, pseudo-randomly arranged in 7 columns, among 315 stimuli on a white background). Two test modalities were used. The conventional modality (CVR) reproduces the Bells Test in the same format (A4) as the PP format. In contrast, the ecological modality (EVR) reproduces the Bells Test within a hemisphere with a 1-m radius. The horizontal field of the hemisphere has a visual angle of 180°, the vertical field a visual angle of 100° (40° down and 60° up). During the task, participants can move their head and trunk freely. Instructions are identical to those for the PP, with the patient given a maximum of 5 min to cross out all the bells. The variables considered for these tests were the same as for the PP test: number of targets found, lateralized difference in targets found, and time taken to complete the test.

*Virtual reality system usability and satisfaction questionnaire.* Patients were invited to complete this questionnaire after the VR session. This questionnaire was based on the System Usability Scale (SUS). The SUS is a commonly used measure to assess the usability of electronic systems, introduced in response to the increasing use of electronic devices in patient management worldwide. Our self-made questionnaire includes 8 items to assess patients’ satisfaction with the VR programs they have experienced. Participants are asked to rate each item on a 4-point response scale ranging from “Strongly disagree” to “Strongly agree”. This questionnaire provides feedback on how the VR experience was perceived by the participants as well as their feelings and emotions.

### Procedure

All patients included in this study performed the PP and the VR Bells Test at a maximum interval of 2 days. During the first session, patients performed the PP bell test following the specific recommendations for this assessment (13). Patients then benefited from a familiarization session with the VR system to try out the HMD and the VR controller. In the second session, patients performed the CVR and EVR modalities of the VR Bells Test. The order of the tests was randomly assigned, and a 5-min rest was imposed between the 2 tests. During the VR Bells Test, patients were seated, wore the HMD attached to their head and held the VR controller in the non-paretic hand. After completing the task, all patients were asked to fill in a questionnaire on usability, satisfaction, and negative effects.

### Data reduction

The variables considered were: (*i*) the number of targets found in each column, (*ii*) the difference between the number of targets found in the 3 right-hand columns and the 3 left-hand columns, and (*iii*) the time taken to complete the test. Based on the cancellation test, USN was determined according to the number of lateralized omissions. A count of 6 or more omissions, or a difference of more than 2 omissions between the left and right sides, was considered indicative of USN (27).

### Data analysis

Statistical analysis was carried out on the whole sample, as well as on each subgroup, i.e., patients with USN and patients without USN. The non-Gaussian distribution of the data was confirmed by the Shapiro–Wilk test. Data were also compared between neglect patients (NP) and no-neglect patients (NNP) using a Mann–Whitney *U* test. Data were compared across the different modalities using a Friedman test or an ANOVA with a post hoc test with a Bonferroni adjusted *p*-value. Intraclass correlation coefficients (ICCs) were used to describe the reliability of the performance results (cancelled bells and lateralized difference in number of cancelled bells) between the 3 modalities. The level of significance for all statistical tests was set to 0.05. All analyses were performed using the software JASP for statistical computing (version 0.18; JASP, Amsterdam, The Netherlands).

## RESULTS

Thirty-two stroke patients were assessed in the study. Participants’ characteristics are presented in Table I. Among these 32 patients, 6 presented a USN according to the clinical observations and the score obtained on the neuropsychological evaluation. The sample (total sample[TS]) was divided into 2 groups: patients without USN (no-neglect patients [NNP], *n* = 26) and patients with USN (neglect patients [NP], *n* = 6) (see Fig. 1).

### Test duration

**Fig. 2:**
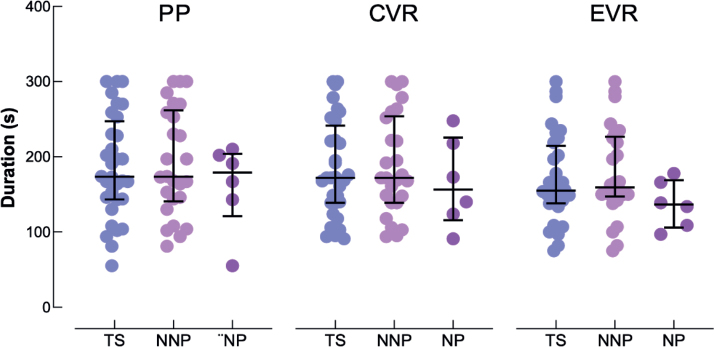
Individual time duration to complete the paper-and-pencil format (PP), and in conventional (CVR) and ecological (EVR) virtual reality format of the Bells Test for all patients (TS, blue discs), no-neglect patients (NNP, pink discs) and neglect patients (NP, purple disks). The large horizontal bars represent the median and errors bars represent the interquartile range.

The time required to complete the Bells Test did not differ between the PP, CVR, and EVR conditions (F[2.62] = 1.324; *p* = 0.273). Results were similar in each subgroup (NNP: F[2.50] = 0.760; *p* = 0.473 ; NP: c^2^ = 4.333; *p* = 0.115) (see Fig. 2).

### Global task performance

**Fig. 3:**
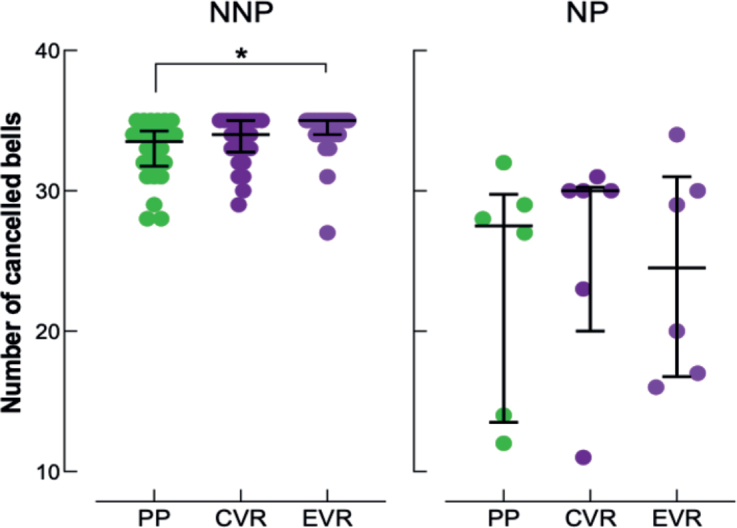
Individual number of cancelled bells in the paper-and-pencil format (PP, green discs), and in conventional (CVR, dark-purple discs) and ecological (EVR, light-purple discs) virtual reality format of the Bells Test: no-neglect patients (NNP) on the right graph, neglect patients (NP) on the left graph. The graph represents the mean standard error and median interquartile. *Denotes a statistical difference between PP and EVR in NNP (Conover post-hoc test: *p* = 0.01).

The reliability between the 3 modalities was high for global task performance (cancelled bells) in the TS (ICC = 0.81, 95% CI, 0.69–0.89). No difference between modalities was noted in the NP (c^2^ = 1.130; *p* = 0.568) whereas a difference was noted in the NNP (c^2^ = 8.674; *p* = 0.013). The post-hoc test shows that this significant difference in the NNP is only between PP and EVR (*p* = 0.01). No difference was noted between PP and CVR (*p* = 0.249) or between CVR and EVR (*p* = 0.554) in the NNP. Results are shown in Fig. 3.

### Lateralized task performance

**Fig. 4:**
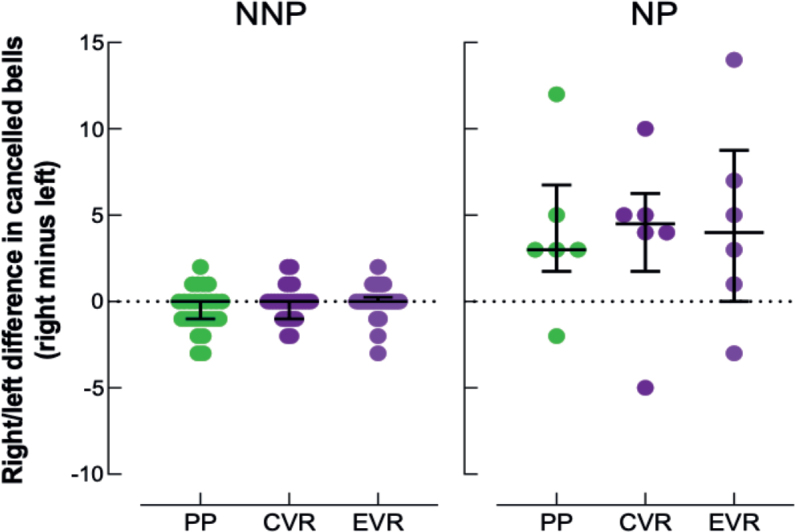
Individual lateralized difference (right minus left side) in number of cancelled bells in the paper-and-pencil format (PP, green discs), and in conventional (CVR, dark-purple discs) and ecological (EVR, light-purple discs) virtual reality format of the Bells Test: no-neglect patients (NNP) on the right side, neglect patients (NP) on the left side. The graph represents the mean standard error and median interquartile.

The reliability between the 3 modalities was high for lateralized task performance (difference right cancelled bells–left cancelled bells) in the TS (ICC = 0.80, 95% CI, 0.68–0.89). Our results showed that the lateralized difference in number of cancelled bells was not influenced by the modality in TS (c^2^ = 1.846; *p* = 0.397). Results were similar in the NNP (c^2^ = 1.826; *p* = 0.401) and NP (c^2^ = 0.273; *p* = 0.873) subgroups (see Fig. 4).

### Comparison between neglect and no-neglect patients

Bells Tests in the 3 modalities revealed the presence of USN, as defined in the literature through a total number of lateralized omissions being greater than or equal to 6, or a difference between left and right omissions being strictly greater than 2, in the 6 NP, except in EVR for 1 patient. Non-parametric analyses showed that there were significantly fewer cancelled bells in NP than in NNP in the 3 modalities (see Table II). In the same way, our results showed a greater lateralized difference in the number of cancelled bells in NP than in NNP in the 3 modalities (see Table II). Results on the test duration did not show any statistically significant difference between NP and NNP, regardless of modality (*p* > 0.05) (see Fig. 2).

**Table II:** Comparison of neglect and no-neglect patients’ task performance

Type		NNP	NP	U	r	*p*-value
Global task performance	PP	33.5	27.5	144.5	0.853	0.001
	CVR	34	30	148.5	0.904	< 0.001
	EVR	35	24.5	146	0.872	< 0.001
Lateralized performance	PP	0	3	23	-0.705	0.007
	CVR	0	4.5	26	-0.667	0.009
	EVR	0	4	29	-0.628	0.012

Values are median (number of cancelled bells).

PP: Paper-and-pencil format; CVR: conventional virtual reality; EVR: ecological virtual reality; NP: neglect patients; NNP: non neglect patients.

### Usability and side effects

In terms of ease of use and the usefulness of the VR in assessment and rehabilitation, over 94% of patients responded positively. Less than 11% of patients found VR programs boring, stressful, or frustrating. Finally, 18% of patients experienced unpleasant sensations when using VR; these sensations were minor and did not require the VR session to be interrupted. Results are shown in Table III.

**Table III:** Results from the questionnaire assessing VR system usability and satisfaction

Question	Strongly disagree	Disagree	Agree	Strongly agree
I quickly learned how to use the VR programs	0 (0)	3 (1)	28 (9)	69 (22)
I enjoy making VR programs	0 (0)	3 (1)	19 (6)	78 (25)
I find VR programs boring	70 (22)	21 (7)	9 (3)	0 (0)
VR programs stressed me out	82 (26)	6 (2)	9 (3)	3 (1)
VR programs frustrated me	85 (27)	6 (2)	3 (1)	6 (2)
I find VR assessment useful	0 (0)	6 (2)	38 (12)	56 (18)
I have unpleasant sensations when using VR	63 (20)	19 (6)	12 (4)	6 (2)
I find the use of VR in my rehabilitation motivating	0 (0)	0 (0)	34 (11)	66 (21)

Values are in percentages (*n*).

## DISCUSSION

The aim of our study was to investigate the feasibility of using immersive VR to assess USN in stroke patients. We first compared the results of the Bells test in the PP format with the VR formats, using two different modalities to assess near and far extrapersonal space in stroke patients. We then assessed the ability of the VR Bells Test to detect USN by comparing the results of the different tests in NP and NNP. Finally, we evaluated the ease of use and patient satisfaction with this type of VR program.

### Comparison of modalities

First, our results showed that there was no significant difference in test duration between the 3 modalities for all patients (TS) and in each subgroup (NP and NNP). These findings underscore the similarity in difficulty between the PP and the VR tests, as previously demonstrated in the literature (17). Indeed, patients did not need more time to complete the same cancellation task, whether performed with PP or with a VR headset and controller. In terms of performance, our results showed that overall task performance was minimally influenced by the modality used. In TS and NNP, no differences were noted between PP and CVR and between CVR and EVR. The only difference noted was the number of cancelled bells between PP and EVR. Nevertheless, the difference between the 2 modalities was only 1 cancelled bell (33 in PP vs 34 in CVR for TS and 34 vs 35 for NNP), a result that does not reach clinical significance (13). Furthermore, no differences were noted in the NP. These results must be interpreted with caution due to the small sample size of the NP. Similarly, no statistical difference was observed in the lateralized difference in the number of barred bells by modality, either in the TS, NNP or NP groups.

While statistical tests show no difference in the number of cancelled bells in neglect patients, descriptive analysis of the results indicates differences between the 3 test modalities for some of them. These results are relatively heterogeneous, and none of the modalities stand out as being easier or more difficult for NP. However, these differences need to be analysed on a larger sample to be interpreted. Different hypotheses may explain these differences. First, USN can affect near-extrapersonal (i.e., within reaching distance of the subject) and far-extrapersonal space (outside of reaching distance). Several studies showed these two spatial entities to be dissociated (5, 15, 33, 34). Conventional PP assessments examine only peripersonal space, whereas VR allows examination for far-extrapersonal space. In NNP, both assessments (that is, PP and VR), should have the same results but in NP results should be different depending on the type of USN. Accordingly, patients with USN show postural deviations and a lack of exploratory head, eye, and hand movements towards the neglected hemispace (5). Conventional PP tests explore only limited information concerning patients’ gaze and search patterns. The EVR modality reproduces the Bells Test in a hemisphere of 1 m radius with a visual angle of 180° in the horizontal field and 100° (40° down and 60° up) in the vertical field. This format implies that the patient must turn his/her head left and right, up and down to explore the entire space where the stimuli are present. The advantage of being able to test the far-extrapersonal space enables VR to be more sensitive to different types of USN. Furthermore, VR assessment may be particularly useful in the chronic stage (i.e., more than 6 months after stroke) when PP methods become less sensitive (35). Moreover, there are also different degrees of USN deficits. Several studies have reported that VR was more sensitive than the conventional PP test in detecting mild USN deficits (4, 20, 23, 36). Finally, some authors suggest that the limited field of view of the HMD encourages participants to focus their attention on the task and may therefore influence the results of the various tests (29).

### Assessing unilateral spatial neglect detection through virtual reality

In the Bells Test, the presence of USN is defined as a total number of lateralized omissions strictly greater than 5 or a difference between left and right omissions strictly greater than 2 (27). Our results in the VR modalities, CVR and EVR, revealed the presence of a USN in the 6 NP, except for 1 patient in EVR. Our results, in terms of number of cancelled bells and the lateralized difference in number of cancelled bells, were significantly different between NP and NNP both in the traditional PP test and in the 2 modalities of the VR test. Several previous studies (28-31) have reported similar results, i.e., poorer performance by NP compared with NNP in several PP cancellation tests, but also in VR. These results support our first hypothesis, namely that the VR Bells Test is at least as effective in detecting USN as the PP Bells Test in stroke patients. However, these results must be interpreted with caution due to the small sample size. Furthermore, our results show that the time taken to complete the cancellation task is identical in both groups (NP and NNP) in all 3 modalities (PP, CVR, EVR). The duration of the Bells Test is capped at 5 min, and the test is automatically terminated once this time limit is reached (13). When performing a cancellation test, a longer duration means greater difficulty in finding all the targets to cancel. Our results show that NP do not seem to have more difficulty than NNP. However, as the total time depends on the number of targets found and the number of targets cancelled is lower in NP, the time per target is *ipso facto* greater in NP. These results confirm that NP need more time to scan a scene (21, 22); this can probably be explained by the more global attentional deficits present in NP (32). One of the advantages of VR is the possibility of analysing numerous parameters calculated automatically by the system, such as time per target or visual exploration behaviour during the task, to provide a more detailed analysis of the strategy used by the patient. Future studies should focus on these different parameters to objectivize the relevant clinical information to be derived from them.

### Usability and acceptance

Our evaluation is that the configuration of the VR system with the chosen cancellation task presents a high degree of usability and satisfaction for the patient, without causing cybersickness-type side effects. With regard to ease of use, previous studies using VR with HMD for the assessment of USN have also found the configuration to be highly feasible (22, 34). Our results confirm this degree of usability, with 97% of our stroke patients, with or without USN, reporting that they quickly learned to use the VR programs, even though none of them had any prior knowledge of VR.

Other studies have shown that VR has a high acceptance rate even in elderly or critically ill patients (36). Several pilot studies have shown similar results in patients with USN (4, 17). In addition, by designing gamified tasks and visually appealing environments, VR can increase patient motivation during assessment or rehabilitation (37). In our study, over 90% of patients enjoyed VR programs and found them useful and motivating. Nevertheless, acceptance may decrease in the event of cybersickness (38). The occurrence of unpleasant sensations was 18% in our sample. However, we did not study the intensity and type of unpleasant sensations reported by patients. The use of a more specific questionnaire, such as the Simulator Sickness Questionnaire (SSQ) (39), would enable more specific analysis of the symptoms of cyberspace sickness (nausea, headache, visual fatigue, dizziness, headaches, and sweating). It is noteworthy that none of the patients, even those who reported unpleasant sensations, asked to interrupt the VR session, suggesting that these symptoms were relatively minor. These results are in line with the literature, which has shown that cybersickness can be caused by sensory conflicts present mainly in virtual programs involving subject movement or scrolling of the landscape (40).

### Conclusion

In conclusion, the results of this pilot study show that the VR cancellation Bells Test is easy to use, very well accepted by patients and does not induce any relevant side effects in stroke patients with or without USN. Furthermore, the similarity of test results across the 3 modalities (PP, CVR, EVR) and the high correlation between them confirm the relevance of the VR test configuration for detecting USN in stroke patients. Overall, this study supports a VR cancellation bell test as a viable alternative to the traditional PP cancellation bell test. Additionally, as pointed out by our descriptive analysis, as VR assesses both near (through CVR) and far extrapersonal space (through EVR), it could be hypothesized that VR would offer a more comprehensive characterization of USN. Further research should focus on comparing CVR and EVR modalities in NP to further investigate the potential contribution of these different modalities to USN assessment.
